# Rapid Repair Epoxy Mortar Fully Replacing Natural Aggregate and Filler with Graded Iron Tailings: Performance and Reinforcement

**DOI:** 10.3390/ma19153324

**Published:** 2026-08-05

**Authors:** Anhua Xu, Jiming Xiao, Yuchang Duan, Huaxin Chen, Jincheng Yu, Fayun Lei, Dongliang Kuang

**Affiliations:** 1 School of Water Resources and Civil Engineering, Qinghai Polytechnic University, Xining 810003, China; xahua@ghmu.edu.cn (A.X.);; 2 Qinghai Provincial Key Laboratory of Tibet Plateau Highway Construction and Maintenance Technology, Xining 810003, China; 3 Qinghai Provincial Traffic Control Construction Engineering Group Co., Ltd., Xining 810028, China; 4 School of Materials Science and Engineering, Chang’an University, Xi’an, 710061, China

**Keywords:** iron tailings, epoxy resin mortar, industrial solid waste, recycled aggregate, pavement rapid repair

## Abstract

Numerous studies have investigated the application of iron tailings as raw materials for cement concrete, while rare efforts have been devoted to developing epoxy pavement repair mortars where graded IT function simultaneously as fine aggregate and filler. This study prepared epoxy resin mortar for rapid repair of cement concrete pavement using graded iron tailings (IT) as fine aggregate and filler. All mixtures employed IT as fine aggregate, where the mass proportion of IT filler relative to the total IT mixture varied from 0% to 40%. The effects of IT filler content on workability, curing temperature, mechanical properties, volume stability, and bond strength were investigated, accompanied by microstructural analysis. Experimental results reveal that the incorporation of IT filler ameliorates the fresh workability and shortens the setting time of epoxy mortar, whereas the compressive strength, flexural strength and interfacial bond strength exhibit an initial ascending followed by a descending trend with the increasing IT filler fraction. The optimal IT filler content is determined to be 20%. For fresh-state performance, the modified mortar delivers a flowability of 176.8 mm (+29.7% relative to EM-0) and a setting time of 127 min. In terms of hardened mechanical and bonding properties, the 28-day compressive strength, 28-day flexural strength and 7-day flexural bond strength reach 113.2 MPa (+17.2%), 42.5 MPa (+34.0%) and 9.1 MPa (+75.0%), respectively, in comparison with the reference specimen EM-0. Microscopic characterizations deliver indirect evidence that appropriate IT filler may refine internal pore-size distribution, densify matrix microstructure and suppress crack propagation. The failure mode changed from pure interfacial debonding to cohesive failure in the cement substrate. This work provides a green scheme for resource utilization of iron tailings in high-performance pavement repair materials.

## 1. Introduction

Iron tailings (IT) are solid industrial waste generated by the mining industry after ore beneficiation and iron extraction [1,2,3]. As the global economy continues to develop, the demand for iron and steel is increasing, leading to a continuous increase in the discharge of iron tailings. Currently, the main method for dealing with iron tailings is to stack them. By the end of 2020, the amount of iron tailings stored in China had exceeded 60 billion tons [4,5]. The large accumulation of iron tailings has brought many problems, such as imposing a heavy financial burden on local governments and causing ecological issues, including pollution of water sources and soil, occupation of construction or agricultural land, and posing safety hazards to the residents around the tailings [6,7,8]. In order to reduce the storage of iron tailings, extensive research has been conducted worldwide on the recycling and utilization of iron tailings, with methods including the recovery of valuable metals [9], as fill materials [10], for concrete coarse and fine aggregates [11], and as raw materials in brick production [12].

The application of building materials is extensive and is one of the main ways to accommodate solid waste. Due to the main components of iron tailings being SiO_2_ and Al_2_O_3_, they have the potential to be used as building materials. Many scholars have used iron tailings as coarse and fine aggregates in cement concrete [13,14]. Oritola et al. [15] found that when iron tailings sand replaced 30% of the fine aggregate, the compressive strength of the concrete containing iron tailings reached its maximum values. Zhao et al. [16] used iron tailings to replace natural aggregates in the preparation of ultra-high-performance concrete. It was found that when the iron tailings replaced no more than 40% of the natural aggregates, the mechanical properties of the concrete were not affected. Kuranchie et al. [17] prepared concrete with a strength grade of 40 MPa by completely replacing natural aggregates with iron tailings sand. The concrete with iron tailings sand had competitive mechanical properties with an 11.56% increase in compressive strength compared to reference concrete, but a 16% decrease in splitting tensile strength. Furthermore, the corrosion resistance and acid resistance of concrete with iron tailings sand were improved. These studies have shown that it exhibits promising feasibility to prepare cement concrete by completely replacing natural fine aggregates with iron tailings.

Although extensive studies have validated the feasibility of using iron tailings in cement-based concrete, research on its application in epoxy resin mortar remains insufficient, especially for pavement repair scenarios. Epoxy mortar exhibits distinct differences from cement concrete in curing mechanism, interfacial behavior, and service requirements. Few studies have focused on the graded utilization of iron tailings as both filler and fine aggregate in epoxy repair mortar, and the influence of filler–aggregate ratio on workability, curing temperature, mechanical properties, bond strength, and volume stability is still unclear. This knowledge gap limits the high-value utilization of iron tailings in high-performance organic repair materials.

Cement concrete pavements often suffer from different levels of diseases such as cracks, pits, and rough surfaces due to vehicle impact and service environment [18]. To ensure traffic safety and extend service life, timely repairs are necessary. At present, there are two main types of repair materials available for cement concrete pavements. The first category is organic repair materials that use high molecular weight materials like epoxy resin. The second category is inorganic repair materials that use fast-hardening cementitious materials such as Portland cement, sulphoaluminate cement, and magnesia phosphate cement [19,20]. Represented by epoxy resin mortar (EM), organic repair materials have been widely used due to their advantages of fast hardening, outstanding mechanical performance, superior durability, high bonding strength, and low shrinkage [21,22,23,24].

A series of studies have proven the feasibility of adopting various industrial solid wastes and recycled aggregates to substitute natural quartz sand and quartz powder in epoxy repair mortar. Lakhiar et al. [25] adopted coal bottom ash as fine aggregate replacement and acquired epoxy mortar with enhanced mechanical strength, impermeability and reduced shrinkage. Bourguiba et al. [26] utilized construction recycled sand; although fresh workability and mechanical strength slightly declined, pore structure and chloride resistance were optimized. Dinberu et al. [27] further confirmed that moderate waste silt filler could boost the elastic modulus and interfacial adhesion of epoxy matrix. Dębska et al. [28] partially substituted epoxy resin with recycled PET derivatives and achieved superior chemical corrosion resistance compared with conventional cement mortar. Therefore, it is feasible to use iron tailings to replace natural sand and fillers to prepare epoxy resin mortar. However, there is little research on the application of iron tailings in the preparation of epoxy resin mortar. Furthermore, the proportion of aggregates and fillers in epoxy mortar has a noteworthy impact on its performance [29], but the reasonable proportion of IT filler and IT sand in epoxy resin mortar remains unclear.

Extensive research efforts have been dedicated to epoxy-based pavement repair mortars regarding solid waste valorization, matrix modification and interfacial bonding characteristics, which lay a solid technical framework for developing epoxy repair composites utilizing recycled iron tailings. Liu et al. [30] fabricated glass fiber–epoxy adhesive-modified cement mortars and systematically elucidated the modulating effects of fiber and resin dosages on the compactness, pore microstructure, mechanical strengths and brittleness of the resultant mortars. Zhang et al. [31] synthesized nano-SiO_2_ epoxy gel mortars synergistically reinforced with hybrid PVA-steel fibers; scanning electron microscopy (SEM) characterization further uncovered the multi-scale synergistic strengthening mechanisms governing workability, mechanical performance, fracture toughness and substrate interfacial adhesion. Combining experimental characterizations with numerical modeling, Hang et al. [32] probed the flexure-shear fracture behaviors of concrete–epoxy mortar interfaces, and clarified the regulatory mechanisms of substrate compressive strength and surface roughness on interfacial fracture energy and mechanical interlocking. Ahmed et al. [33] proposed a composite strengthening strategy integrating epoxy injection, shrinkage-compensated micro-mortar and externally bonded carbon fiber-reinforced polymer (CFRP) laminates for rehabilitating deteriorated bridge girders, whose structural reinforcement efficacy was validated via full-scale field load testing. A research group from the Qingdao University of Technology [34] modified ordinary Portland cement repair mortars with waterborne epoxy resins, and verified that appropriate epoxy incorporation could substantially enhance flexural toughness and diverse interfacial bonding strengths without compromising compressive resistance. Rajeshwar et al. [19], Dalhat [35] and Maherzi et al. [36] separately introduced sand washing residues, date pit wastes and dredged sediments as alternative aggregates for epoxy mortars, and quantitatively analyzed the dose-dependent evolution of drying shrinkage, thermal expansion coefficient and mechanical properties induced by various solid waste fillers. These preceding studies demonstrate that industrial solid wastes are promising alternatives to natural aggregates for manufacturing epoxy repair mortars, where the filler-to-aggregate mass ratio acts as a decisive parameter governing fresh workability, mechanical robustness, dimensional stability and interfacial bonding performance. Nevertheless, prior investigations rarely involve the graded utilization of iron tailings. Systematic investigations on epoxy repair mortars with iron tailings powder and iron tailings sand simultaneously serving as filler and fine aggregate are still lacking, and the intrinsic mechanism linking the mass fraction of iron tailings filler to the comprehensive performance of epoxy mortars remains unclear.

To fill this knowledge gap, this study completely replaced natural fillers and aggregates with screened iron tailings powder and iron tailings sand, respectively, to prepare epoxy resin mortar. The research examined how varying the ratio of iron tailings fillers to fine aggregates impacted work performance, internal temperature, mechanical properties, length changes, and bonding strength of epoxy resin mortar. The microstructure of epoxy resin mortar with iron tailings was examined by scanning electron microscopy, mercury intrusion porosimetry and digital image correlation method. The reinforcing mechanism of iron tailings in the epoxy resin was analyzed. The utilization of iron tailings as filler and aggregate to prepare epoxy resin mortar could recycle mining waste resources. The prepared material is intended for use as a rapid repair material for damaged cement concrete pavements. Epoxy repair mortars used for concrete pavement rehabilitation are generally required to meet the EN 1504-3 R4 class for structural repair materials, which specifies minimum thresholds for compressive strength, flexural strength, and interfacial flexural bond strength.

The core scientific hypothesis of this investigation is proposed as follows. Graded iron tailings with dual particle sizes can simultaneously act as fine aggregate and mineral filler inside epoxy repair mortar. When an appropriate mass fraction of iron tailings filler is incorporated, the composite particle packing structure can be optimized, internal pore defects are refined, and crack deflection and particle toughening effects are triggered. Such synergistic effects are expected to improve fresh workability, mechanical strength, dimensional stability and interfacial bonding performance between the repair mortar and cement substrate. Nevertheless, excessive iron tailings filler will cause severe particle agglomeration, introduce extra air voids, and hinder the crosslinking curing reaction of epoxy resin and curing agent, eventually leading to degradation of overall material performance. This hypothesis will be comprehensively verified via macroscopic performance tests and multi-scale microscopic characterizations including SEM, MIP and DIC.

## 2. Materials and Methods

### 2.1. Materials

**Figure 1:**
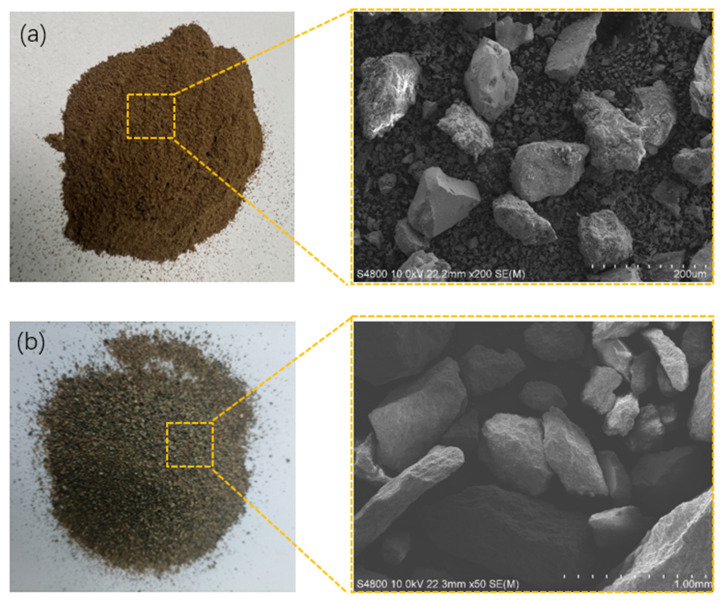
SEM morphologies of IT filler and IT fine aggregate; (**a**) filler; (**b**) aggregate.

**Figure 2:**
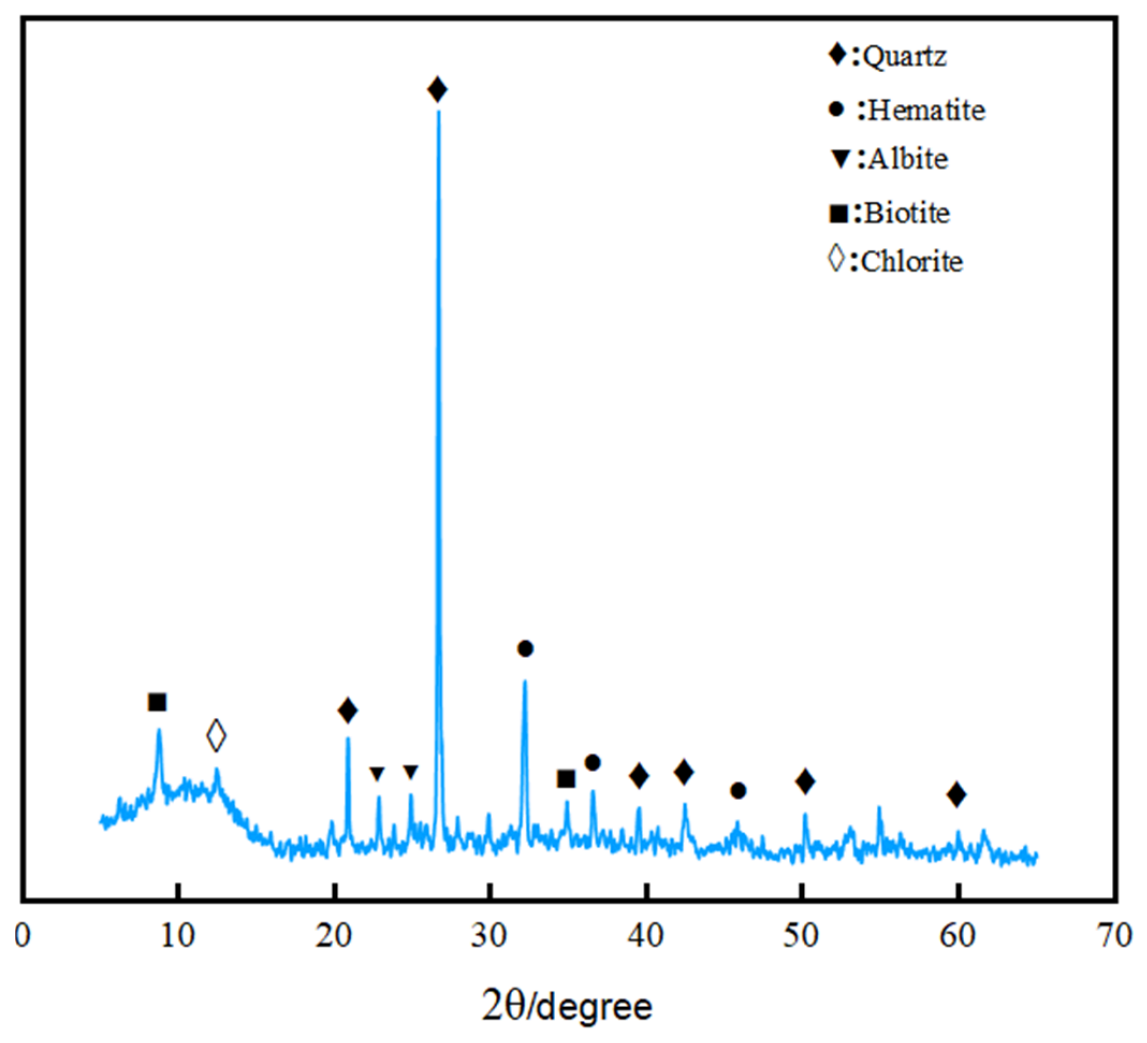
XRD image of iron tailings.

The bonding material used in this experiment was a mixture of epoxy resin, curing agent, and diluent. The epoxy resin E-51, curing agent 593 and diluent 692 were used in this study. Epoxy resin E-51 was bisphenol epichlorohydrin with epoxide equivalent of 184~194 g/eq. The curing agent 593 was a mixture of diethylenetriamine and benzyl alcohol, while the 692 diluent was benzyl glycidyl ether. The fundamental physicochemical properties of E-51 epoxy resin, 593 curing agent, and 692 reactive diluent are summarized in Table 1, Table 2 and Table 3. The iron tailings were obtained from the second Cigou tailings dam of Shaanxi Daxigou Mining Co., Ltd. (Shangluo, China), and the chemical elemental and morphology of the iron tailings are shown in Table 4 and Figure 1, respectively. The primary mineral components found in iron tailings are quartz, hematite, albite, and biotite. Before mixing, the iron tailings were dried at 105 °C for 24 h to remove free water, then mechanically crushed and graded by standard sieves. The iron tailings were strictly divided into two fractions: particles with a size of 75 μm–1 mm were used as IT fine aggregates, and particles smaller than 75 μm were used as IT fillers. The grading process ensured that the aggregate fraction contained no particles smaller than 75 μm. Before preparing epoxy resin mortar, the iron tailings were sieved and classified according to the above particle size standards. The macro- and micro-morphologies of the iron tailings fine aggregates and fillers are depicted in Figure 2. There are more edges and corners on the surface of iron tailings. The substrate mortar used in the bond strength test was ordinary cement mortar, composed of P·O 42.5 Portland cement and standard sand.

### 2.2. Experimental Design

The range of IT filler content (0–40 wt.% of total iron tailings) and 10% interval gradient were determined through preliminary trial mixing. Pre-experiments revealed that fillers below 10% failed to deliver obvious particle packing optimization, while filler dosage exceeding 40% triggered severe particle agglomeration, poor flowability and drastic strength loss. Thus, 0%, 10%, 20%, 30% and 40% were selected as representative mixing ratios to capture the full variation trend of mortar performance. In this experiment, EM-0 was defined as the control group, in which iron tailings were used only as fine aggregates without any iron tailings filler. All mixtures shared identical dosages of epoxy resin, curing agent and reactive diluent, and the total mass of iron tailings (filler + fine aggregate) remained constant. No extra water was incorporated throughout the mixing procedure of iron tailings-modified epoxy mortar. The detailed mix proportions of epoxy mortars with different IT filler fractions are listed in Table 5. The mix proportions were determined based on pre-experiments and particle grading optimization. All mixtures used the same amount of epoxy resin, curing agent, and diluent. The total mass of IT (filler + fine aggregate) was kept constant. The label “EM-X” means that IT filler accounts for X% of the total IT mass by weight. For example, EM-0 represents 0% IT filler and 100% IT fine aggregate; EM-20 represents 20% IT filler and 80% IT fine aggregate by total IT mass. In this study, iron tailings (IT) were fully used as fine aggregates in all mixtures, and graded IT powder (<75 μm) was used as filler. The variable was the mass percentage of IT filler in total IT (filler + fine aggregate). EM-0 was the control group with 0% IT filler and 100% IT fine aggregate.

### 2.3. Specimen Preparation and Test Method

#### 2.3.1. Specimen Preparation

The corresponding raw materials were weighed according to the designed mix ratio. Then, the epoxy resin, curing agent and diluent were poured into a mixing pot and stirred with an electric mixer for 60 s. Next, the iron tailings fillers were added and stirred for another 60 s. Finally, the iron tailings fine aggregates were added and mixed for 180 s to obtain the epoxy mortar.

Subsequently, the prepared epoxy mortar was poured into a three-part mold size of 40 mm × 40 mm × 160 mm, in two layers. After each pouring, the mold was placed on a vibrating table to compact the mortar and then leveled with a scraper. After molding, the specimens were placed in a curing box at a temperature of 20 °C ± 2 °C and a relative humidity of 50% ± 5% for 24 h. Then, they were demolded and further cured under the same environment until the desired testing age. The specimen preparation, mixing, casting, vibration compaction and curing procedures were carried out in accordance with GB/T 2567-2021 [37], JC/T 2630-2021 [38] and GB/T 17671-2021 [39].

Three groups of specimens with different dimensions were fabricated for corresponding tests: 40 mm × 40 mm × 160 mm prisms for flexural, compressive and flexural bond strength tests; 25 mm × 25 mm × 280 mm prisms pre-installed with measuring nails at two terminals for volume stability measurement; and small cubic fragments of around 10 mm × 10 mm × 10 mm cut from fractured samples for SEM and MIP microstructural analysis.

#### 2.3.2. Test Method

All tests were carried out in chronological order and split into two categories: fresh-state tests (flowability, setting time and internal curing temperature) and hardened-state performance tests (flexural strength, compressive strength, volume stability and interfacial bond strength).

**Figure 3:**
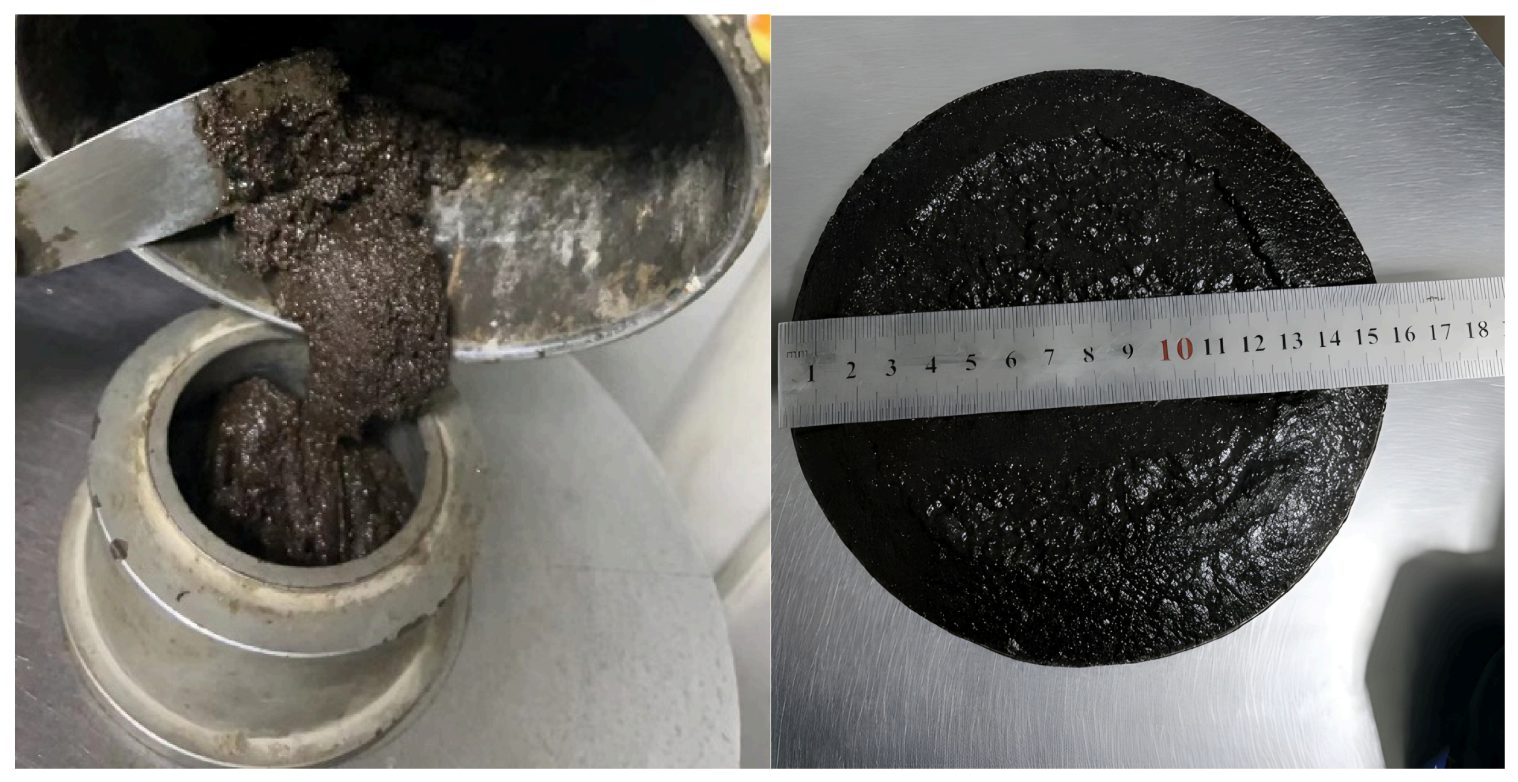
The flowing table test (unit of mm).

(1) Flowability test: The flowability of epoxy resin mortar was tested in accordance with the Chinese standard GB/T 2419-2005 [40]. After the mortar stopped spreading on the flow table, the maximum spreading diameter and the perpendicular transverse diameter were separately measured. The arithmetic average of these two mutually perpendicular diameters was recorded as the final flowability value. The experimental procedures are shown in Figure 3.

(2) Setting time test: The setting time of epoxy resin mortar was measured using a Vicat apparatus, referring to the standard GB/T 1346-2011 (China) [41].

(3) Internal reaction temperature test: The internal temperature of the epoxy mortar was measured using a temperature-rise recorder, temperature sensors and software. The temperature-rise test was conducted in a curing room with a temperature of 20 °C ± 2 °C. First, the temperature probe of the temperature sensor was placed in a previously prepared beaker. The temperature recorder was immediately turned on after 193.5 g of epoxy resin mortar was quickly poured into the beaker. The test ended after 8 h.

(4) Flexural and compressive strength tests:

Both flexural strength and compressive strength were determined with a SANS universal testing machine (SANS, Shenzhen, China). Flexural strength was tested at 3 d, 7 d, and 28 d according to GB/T 17671-2021. The flexural strength specimens were formed in a three-piece mold with dimensions of 40 mm × 40 mm × 160 mm. The specimens, cured to a specific age, were placed in the universal testing machine equipped with the flexural fixture. The loading rate was set at 50 N/s. After the specimens were fractured, the ultimate failure load was recorded and the average value of the three specimens was taken as the flexural strength value.

Compressive strength was tested at 3 d, 7 d, and 28 d according to GB/T 17671-2021 [39]. The compressive fixture was installed on the universal testing machine, with a loading speed of 2.4 kN/s. Mortar specimen size was kept at 40 mm × 40 mm × 40 mm. The ultimate load was recorded after the specimens were fractured and three specimens were tested for each group to obtain the average value.

(5) Volume stability test:

The volume deformation of epoxy resin mortar referred to the experimental method for drying shrinkage of cement mortar in JC/T 603-2004 [42]. The length of specimens was measured right after demolding (initial length reference), followed by regular detection at curing ages of 3 d, 7 d, 14 d, 28 d and 60 d. All dimensional measurements were finished at a constant ambient temperature to eliminate thermal expansion interference. Triple molds with dimensions of 25 mm × 25 mm × 280 mm were used to form the specimens, with dry shrinkage measurement nails pre-set at both ends. After 24 h of mold removal, the specimens were cured at an ambient temperature of 20 °C ± 2 °C and relative humidity of 50% ± 5%. After determining the initial length, the subsequent measurements were taken at specified ages to calculate the volume deformation rate of the specimens according to the following equation:

(1) $$\begin{array}{c} d_{i}=\frac{10000\left(L_{i}-L_{0}\right)}{L}\times 10^{-4} \end{array}$$

where d_i_ represents the shrinkage rate of the specimens at specific ages; L_i_ denotes the length of the specimen at a certain age in millimeters; L_0_ denotes the initial length of the specimen in millimeters; and L denotes the effective length of the specimen, which is taken as 250 mm. The shrinkage rate values are the average of three specimens. The multiplier of 10^−4^ is adopted to convert the calculated strain value into an intuitive order of magnitude. The shrinkage deformation of epoxy mortar is tiny, and direct strain values are often at the magnitude of 10^−4^. This coefficient avoids frequent decimal zeros and facilitates data reading, comparison and statistical analysis.

**Figure 4:**
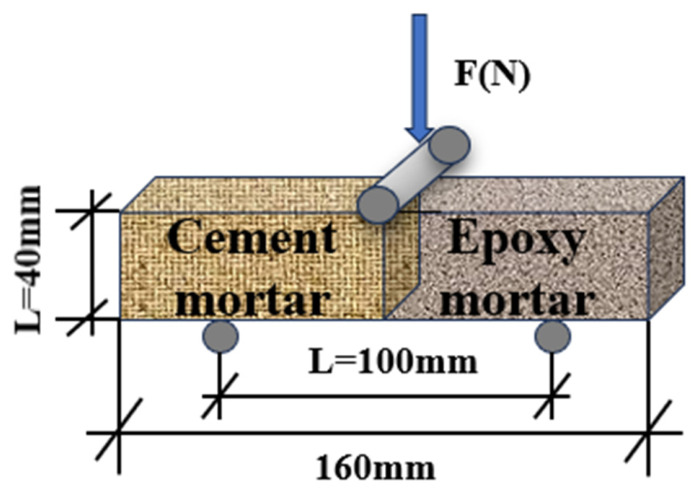
Bond strength test.

(6) Bond Strength Test:

Bond strength is a key index for pavement repair materials, reflecting the interfacial adhesion between the repair material and the old concrete substrate. The bond strength was evaluated using the flexural bond strength test method. The test specimens comprised a composite of half old cement mortar and half epoxy resin mortar, with total dimensions of 40 mm × 40 mm × 160 mm according to GB/T 50728-2023 [43]. The half-cement mortar block was obtained by cutting a 28-day cured cement mortar under standard curing conditions. The epoxy resin mortar half-block was directly poured close to the precast cement mortar half-block in the mold, as illustrated in Figure 4. After pouring, the specimens were cured following standard conditions over 7 days for testing. Subsequently, the specimens were placed in a universal testing machine with a loading speed set at 50 N/s. The ultimate load was recorded and the bond strength value was calculated as the average of three specimens.

(7) Scanning electron microscopy analysis:

Samples taken from the fracture surface of the flexural strength test specimens were sputter-coated with gold and then observed under a scanning electron microscope. The scanning electron microscope utilized was the S-4800 field emission scanning electron microscope (SEM) (Hitachi, Tokyo, Japan), operating at an 8 kV working voltage and a work height of 25 mm. The attached energy-dispersive X-ray spectroscopy (EDS) detector was employed for elemental scanning at specific points.

(8) Mercury intrusion porosimetry (MIP) test:

Mercury intrusion porosimetry was used to characterize the pore structure of hardened mortar. The test instrument operated with an intrusion pressure ranging from 0.003 MPa to 220 MPa. The sample volume was controlled to be approximately 10 mm × 10 mm × 10 mm.

(9) Digital image correlation method:

The deformation behavior of the bonding interface between epoxy resin mortar and old cement mortar with different tailings filler content was investigated by the digital image correlation (DIC) testing method. The Vic-2D non-contact full-field two-dimensional strain measurement system, manufactured in the United States, was employed in the experiments, which mainly consists of a high-speed CCD camera, lighting system and computer. During the tests, real-time capturing of the bonded specimens was performed using the CCD camera and the speckle patterns captured before and after specimen fracture were analyzed using the VIC-2D software (version 2019) (Correlated Solutions, Columbia, SC, USA). Strain and displacement changes in different directions during the deformation process of the specimens were obtained through data processing and analysis.

One-way ANOVA was adopted to evaluate the statistical difference in interfacial bond strength among different groups. The independent comparison factor was the types and dosage of modified additives. Each experimental group contained three parallel replicate specimens. The significance level was set as *p* < 0.05. ANOVA was performed on the premise that data conformed to normal distribution and homogeneity of variance. Tukey’s HSD post hoc test was used for pairwise comparison between different groups. All statistical calculations were completed using Origin 2021 software (OriginLab Corporation, Northampton, MA, USA).

ANOVA was only conducted for bond strength instead of flowability, mechanical strength, volume stability and pore structure. The reasons are listed as follows. Bond strength is highly sensitive to tiny changes in interface microstructure and component dosage, and experimental fluctuation easily causes ambiguous trend; statistical discrimination is necessary to confirm whether property differences originate from material modification rather than random experimental error. Other indicators show obvious monotonic change trends with admixture content, and regular variation law can be clearly judged by mean values. Additional ANOVA brings redundant calculation and does not improve the reliability of data interpretation.

## 3. Results and Discussion

### 3.1. Workability

#### 3.1.1. Slump Flow

**Figure 5:**
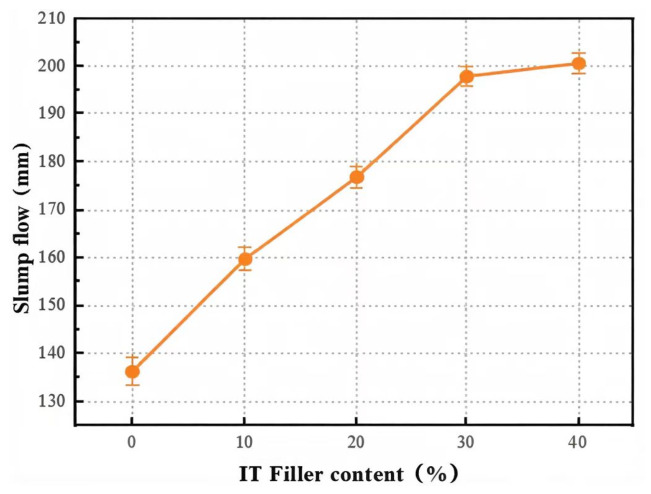
Effect of iron tailings filler content on slump flow of EM.

The influence of iron tailings filler content on the flowability of epoxy mortar is shown in Figure 5. As the iron tailings filler content increased from 0% to 30%, the flowability of the epoxy mortar increased significantly. When the content of iron tailings fillers continued to increase to 40%, the flowability remained relatively stable. Without iron tailings filler content, the mortar with only iron tailings fine aggregates had the lowest flowability, with a value of only 136.3 mm. At this point, the mortar was highly viscous and had poor workability for construction. When 20% of the iron tailings fillers were added, the flowability increased by 29.7% compared to the control group. This was because the shape of the iron tailings fillers was irregular and the surface was rough, resulting in significant friction between particles, which led to low flowability in the control group. However, after adding the iron tailings fillers, the smaller particle size and fine particles of the filler acted as rolling lubricating particles between the aggregates, significantly improving the flowability of the epoxy mortar [44].

Compared with coal ash and recycled sand fillers, angular iron tailings achieve better pore filling yet weaker lubrication, leading to slowed flow growth above 30% filler content.

#### 3.1.2. Setting Time

**Figure 6:**
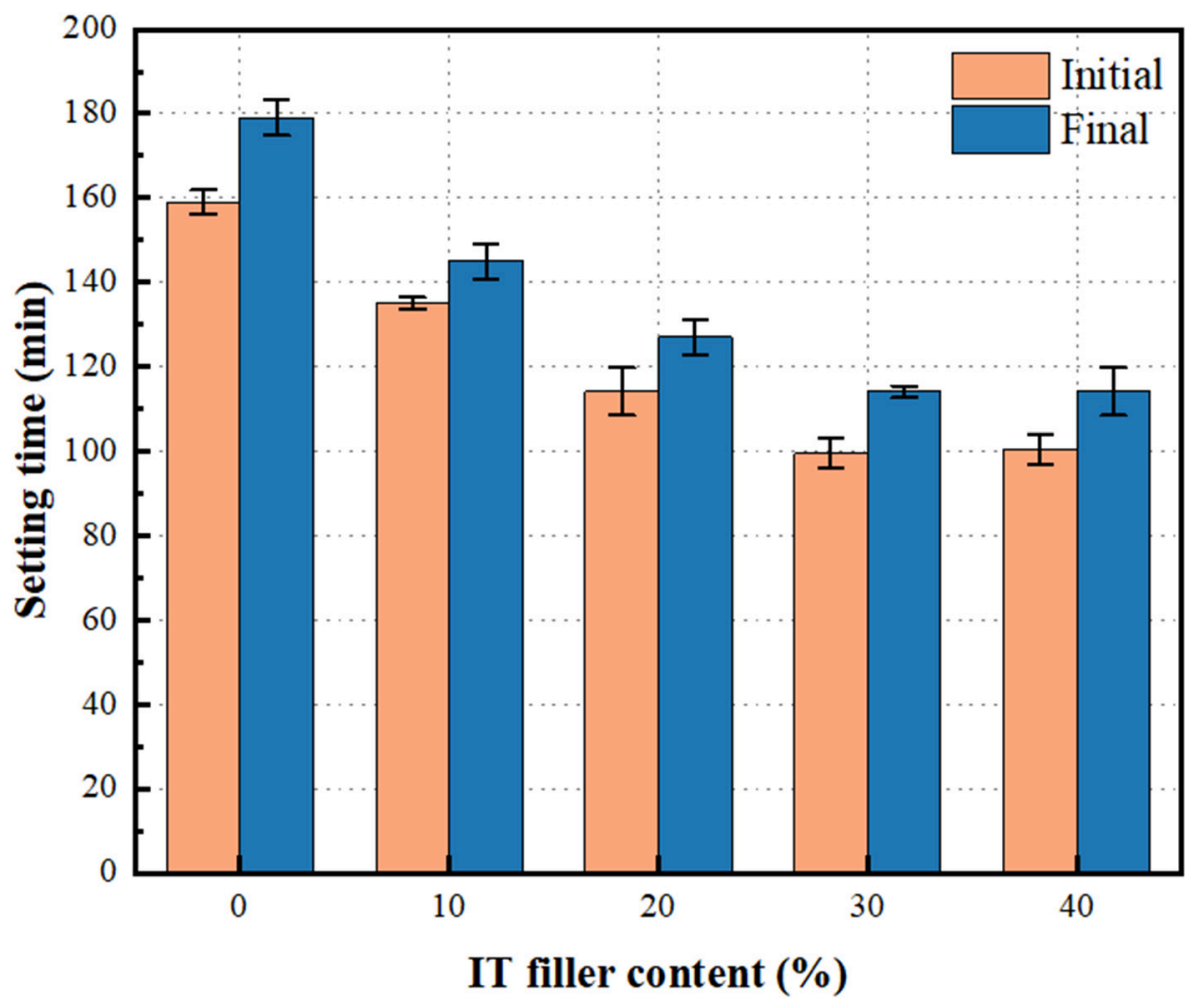
Effect of iron tailings filler content on setting time of EM.

In actual engineering applications, the repair construction time should be as short as possible to open traffic and reduce the losses caused by traffic closures. As shown in Figure 6, the influence of iron tailings fillers and fine aggregates on the setting time of epoxy mortar is depicted. It could be observed from the graph that with the continuous increase in filler content, both the initial and final setting times of the mortar showed a decreasing trend. The control group exhibited the longest setting time at 179 min. When the iron tailings filler content was 10%, the setting time of the epoxy mortar decreased significantly to 145 min. When the filler content was 30%, the setting time decreased to 114 min, which still met the requirements of repair materials. The reason for the shortened setting time may have been that the addition of iron tailings filler increased the compactness of the mortar, leading to an increase in resin density around the aggregate per unit volume, accelerating the curing reaction and reducing the setting time. Fine mineral particles accelerate epoxy crosslinking, shortening setting time as reported by Dinberu et al. [27].

#### 3.1.3. Internal Temperature

Epoxy mortar was formed by the chemical reaction between epoxy resin and the curing agent, resulting in a three-dimensional network structure encapsulating the aggregate. Like the hydration reaction of cement mortar, the curing reaction of epoxy resin and the curing agent was also an exothermic reaction. The heat released by the curing reaction promoted further reactions between the epoxy resin and the curing agent. The present experiment investigated the influence of iron tailings filler content on the internal heat effects of the mortar by measuring the temperature changes inside the mortar.

**Figure 7:**
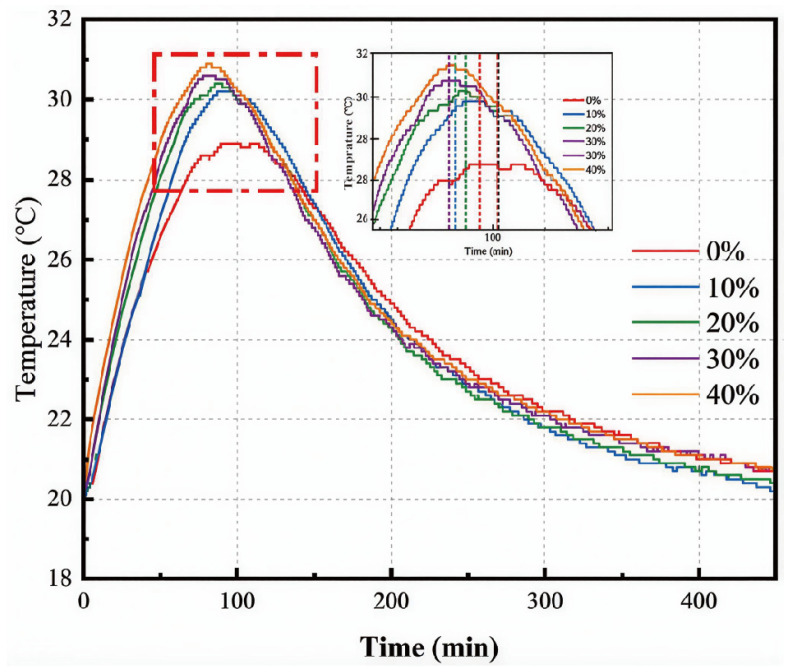
Effect of iron tailings filler on the internal temperature of EM.

The influence of iron tailings filler content on the internal heat effects of epoxy resin mortar is shown in Figure 7. It could be observed that the epoxy resin mortar with different iron tailings filler content exhibited only one exothermic peak. With increasing filler content from 0% to 40%, the peak temperature reached by the epoxy mortar during curing continuously rose, with peak temperatures of 28.9, 30.2, 30.4, 30.6, and 30.9 °C, respectively. The time to reach the peak temperature also increased. Approximately 7 h after the start of the curing reaction, the internal temperature of the mortar returned to the ambient temperature, and the subsequent curing reaction became slow. To some extent, this explained the reason for the shortened setting time of the epoxy mortar. The internal temperature variation is a macroscopic result of curing heat release, matrix thermal conductivity, and heat storage, which cannot be regarded as sole proof of accelerated chemical curing kinetics.

### 3.2. Mechanical Property and Volume Changes

#### 3.2.1. Flexural Strength

**Figure 8:**
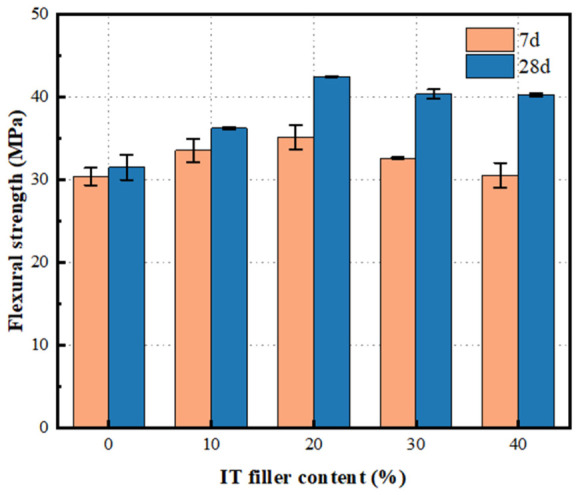
Effect of iron tailings filler content on flexural strength of EM.

The influence of filler and fine aggregate content on the flexural strength of epoxy mortar is shown in Figure 8. It was observed that with an increase in iron tailings filler content, the flexural strength of the epoxy mortar at the same curing age first increased and then decreased, following a similar trend to the compressive strength. The flexural strength was the highest at an iron tailings filler content of 20% for different curing ages, reaching 35.16 MPa at 7 d and 42.5 MPa at 28 d, representing increases of 16% and 34% compared to the control group, respectively. This was because the filler particles of the iron tailings played a “nailing” role in the epoxy mortar, hindering the propagation of cracks when the mortar fractured. When cracks propagated to the interface between the filler and resin, the cracks bypassing the particles required more energy, enhancing the ability of the resin matrix to resist crack propagation and consequently endowing the epoxy mortar with higher toughness [45].

#### 3.2.2. Compressive Strength

**Figure 9:**
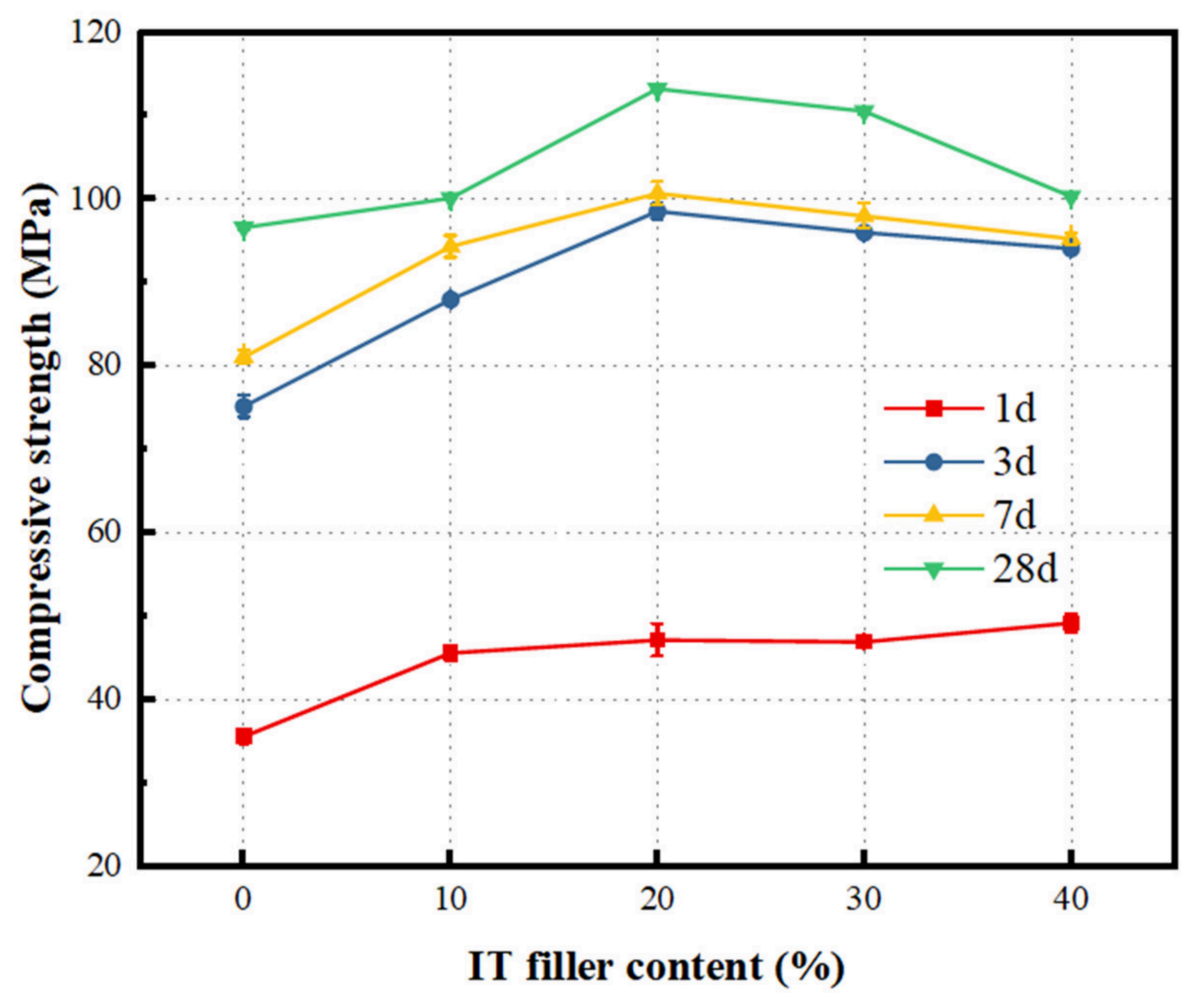
Effect of iron tailings filler content on compressive strength of EM.

The relationship between compressive strength and iron tailings filler and fine aggregate content of epoxy mortar prepared with iron tailings is shown in Figure 9. With increasing filler content, the compressive strength of the epoxy mortar at 1 d increased continuously, while the compressive strength at 3 d, 7 d and 28 d initially increased and then decreased. Overall, the most favorable comprehensive parameters of the epoxy mortar were achieved with an iron tailings filler content of 20%. Without the addition of iron tailings fillers, the compressive strength of the epoxy mortar was only 35.6 MPa at 1 d, which increased to 75.2 MPa at 3 d and showed minimal improvement at 7 d, reaching 81.1 MPa. The compressive strength reached 96.6 MPa at 28 d. This was because with longer curing time, continuous crosslinking reactions occurred between the epoxy resin and curing agent in the mortar, forming a denser and more complete network structure. With the addition of iron tailings filler, the compressive strength of the epoxy mortar increased significantly, reaching its highest value at a 20% filler content. The compressive strength at 3 d reached 94.4 MPa, which was an increase of 25.5% compared to the control group, and reached a peak of 113.29 MPa at 28 d, a 17.2% increase compared to the control group. The reason for this was that the difference in particle size between the filler and aggregate particles was significant. The proper content of iron tailings fillers resulting in a higher number of fine particles per unit volume, making the “bridging effect” more pronounced. This also made the mortar denser, reducing porosity and increasing compressive strength [46]. When the iron tailings filler content exceeded 20%, the strength of the mortar decreased. This was because excessive filler content hindered the contact between the resin and curing agent, leading to a lower degree of curing and a decrease in compressive strength. Unlike cement hydration matrix, excess fine IT powder blocks epoxy curing reaction and reduces strength.

The overall strength evolution trend observed in this work is consistent with the general law reported by Lakhiar et al. [25] and Dinberu et al. [27], who confirmed that moderate solid waste filler can strengthen epoxy matrix while overdosage leads to performance degradation. However, the optimal filler fraction of 20% obtained in this study differs from the 30% optimum replacement rate summarized in cement-based concrete systems. Such discrepancy mainly originates from two core differences in matrix characteristics. On one hand, cement-based materials rely on hydration reaction for strength development, while epoxy mortar’s mechanical performance depends on the crosslinking network of organic resin; fine tailing powder imposes more severe interference on epoxy curing at high dosage. On the other hand, most previous investigations treated iron tailings as a single type of aggregate without particle grading separation, while this study separates tailings into dual-functional coarse aggregate and fine filler fractions to realize synergistic particle packing.

#### 3.2.3. Volume Changes

After the formation of epoxy mortar, certain volume deformations occurred, including inherent volume changes in the reaction products before and after curing, thermal expansion due to exothermic reactions, and shrinkage resulting from cooling. The volume stability of repair materials influenced the tightness of the bond between the repair material and the cementitious substrate. Excessive shrinkage could lead to internal stresses at the interface, causing interface cracking and the risk of detachment of the repair material.

**Figure 10:**
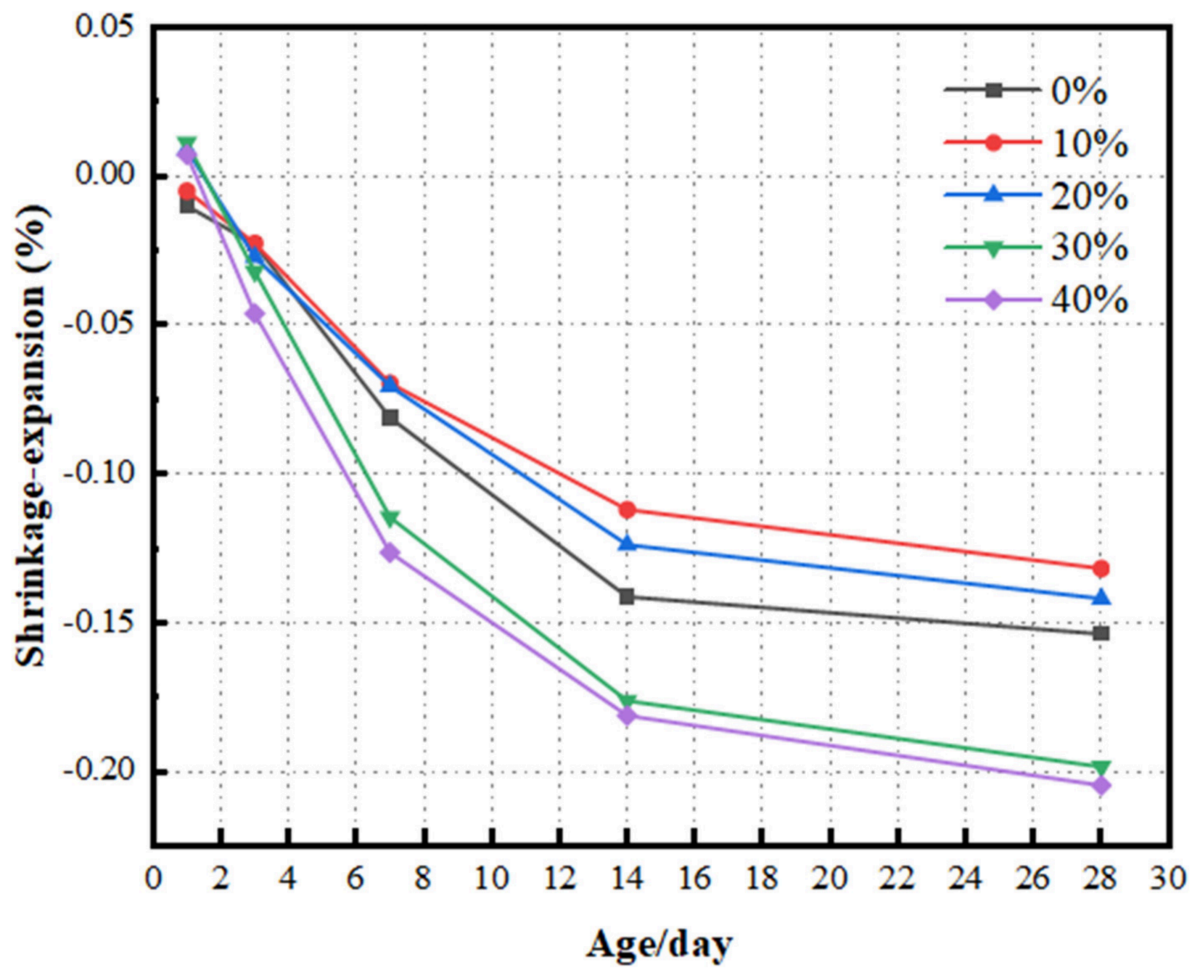
Effect of iron tailings filler content on drying shrinkage of EM.

The effect of iron tailings fillers and fine aggregates on the dimensional stability of epoxy mortar is illustrated in Figure 10. As the content of iron tailings fillers increased, the volume deformation of the epoxy mortar first decreased and then increased. The addition of an appropriate amount of iron tailings filler reduced the shrinkage rate of the mortar. When the iron tailings filler content was 10%, the shrinkage rate of the epoxy mortar was minimum. When the iron tailings filler content was 20%, the deformation magnitude of the epoxy mortar slightly decreased. However, when the iron tailings filler content exceeded 20%, the shrinkage rate of the mortar increased, even exceeding that of the control group. This was because the proper filling of the iron tailings fillers between the epoxy resin and fine aggregate increased the compactness of the epoxy mortar. Additionally, the iron tailings filler was uniformly dispersed in the epoxy resin, which hindered the shrinkage of the resin and reduced the shrinkage rate of the epoxy mortar [47]. Moderate IT filler restrains drying shrinkage, whereas overdosage causes particle agglomeration and larger shrinkage.

### 3.3. Bond Behavior

The four-point flexural bond test specified in GB/T 50728-2023 [43] was adopted in this work, which is consistent with the specimen configuration and loading mode defined in EN 1504-3 [48] for structural repair materials. Compared with direct tensile bond tests, this flexural method can intuitively capture interfacial crack propagation and failure modes, and matches the actual bending stress state of pavement repair layers under vehicle loads; hence, it was selected for interfacial bond characterization.

**Figure 11:**
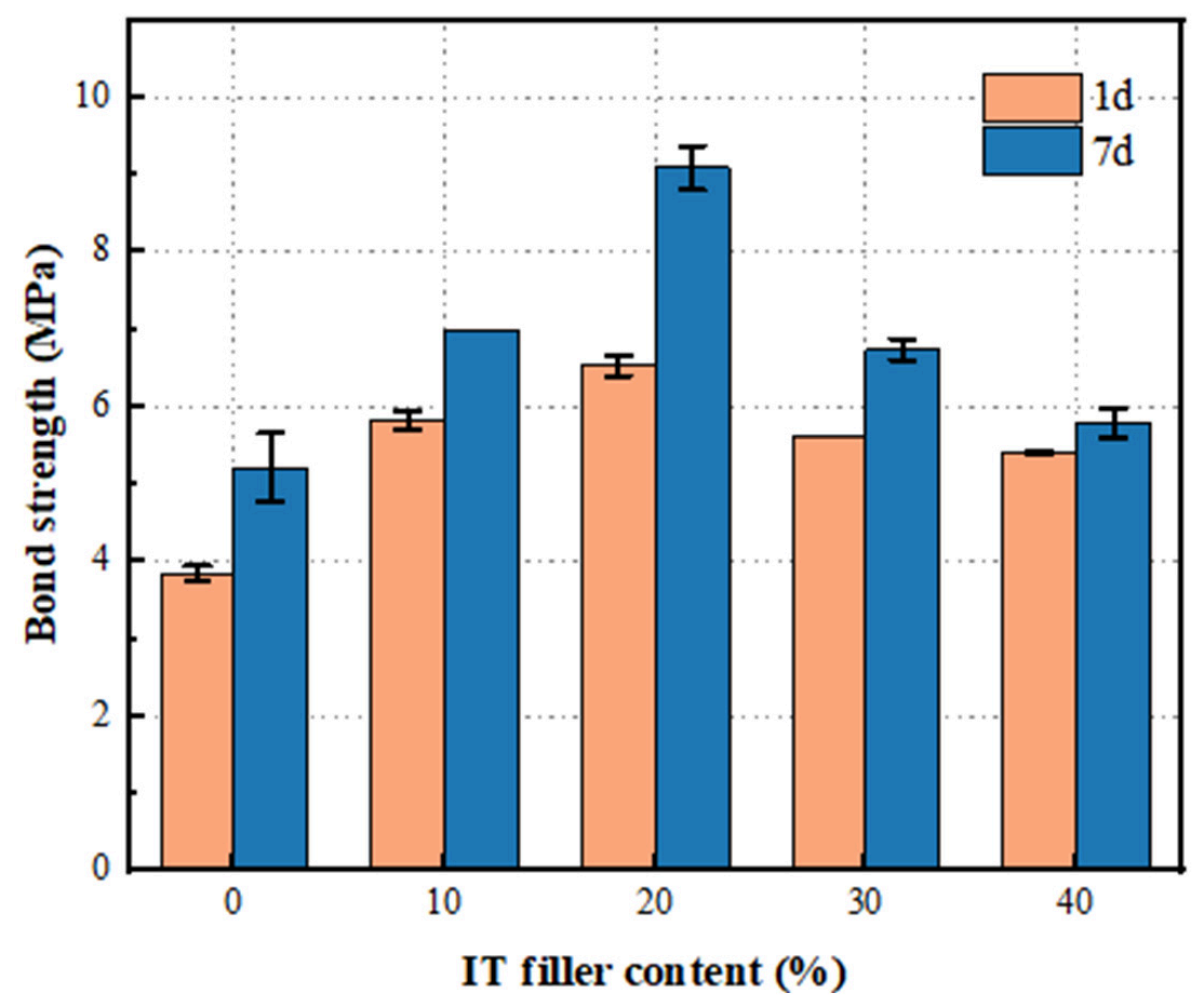
Effect of filler on bond strength of EM.

Significant differences (*p* < 0.05) are detected between specimens with filler content below 20% and above 20% according to ANOVA results, confirming that excessive tailing filler imposes statistically adverse impacts on bonding performance. When repairing concrete pavement, the bond strength between the repair material and the substrate concrete was critical. The effect of different filler contents on the flexural bond strength of epoxy mortar is investigated in Figure 11. The experimental results indicated that the addition of iron tailings fillers significantly enhanced the bond strength of the epoxy mortar compared to the control group, and the bond strength showed an initial increase followed by a decrease as the filler content increased. When the iron tailings filler content was 20%, the bond strength of the epoxy mortar reached its peak, measuring 6.5 MPa at 1 d and 9.1 MPa at 7 d, representing increases of 69% and 75% compared to the control group. As the iron tailings filler content continued to increase, the bond strength began to decrease, but it still remained higher than that of the control group. EM-20 exhibits higher bond strength than traditional quartz powder epoxy mortar. Angular IT particles create stronger mechanical interlocking with cement matrixes, shifting failure from interface separation to substrate cohesive damage, which is an advantage of graded iron tailings.

The reasons for this trend were twofold. Firstly, the poor flowability of the control group mortar led to the generation of numerous defects during the molding progress. Secondly, the larger particle size of the iron tailings fine aggregates in the control group without fillers resulted in a reduced contact area with the substrate concrete, leading to decreased adhesion of the repair mortar. With the addition of iron tailings fillers, the flowability of the mortar was greatly improved, enabling the epoxy mortar to wet and penetrate the rough surface and pores of the substrate mortar. The solidification of epoxy resin in these rough surfaces and pores could generate interlocking and mechanical anchoring effects, ultimately resulting in higher bond strength [49].

**Figure 12:**
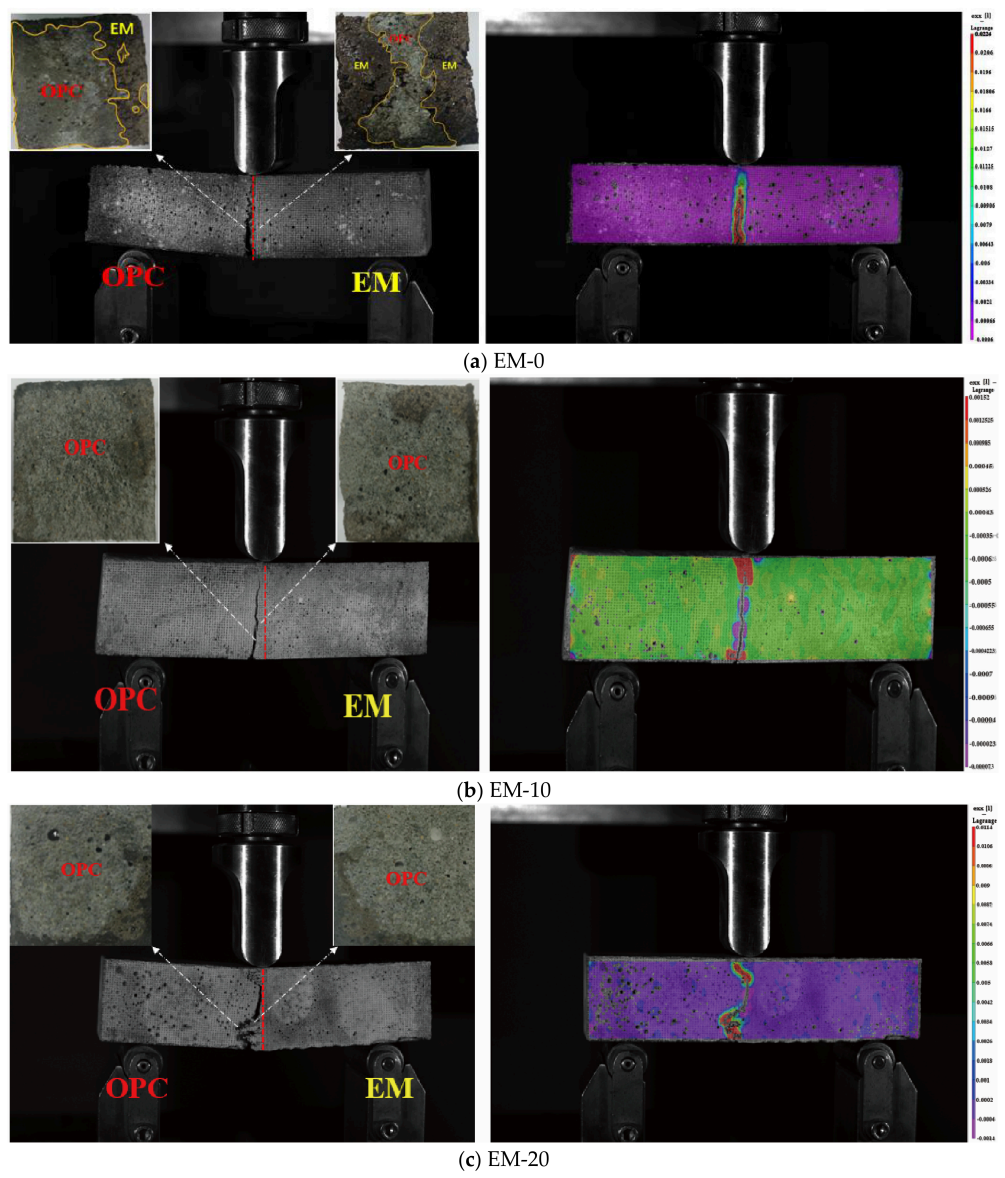
Distribution of bending bond failure and *X*-axis stress between epoxy mortar and cement mortar with different content of iron tailings fillers.

The mechanism of bonding failure and the distribution of *X*-axis stress in epoxy mortars with different iron tailings filler contents are shown in Figure 12. Compared with the reference group without iron tailings fillers, the fracture crack of epoxy resin mortar was inhibited after the addition of iron tailings fillers and was no longer damaged along the interface. The inhibition effect was obvious when the iron tailings filler content in epoxy mortars was 20%. In Figure 12a, the fracture crack of the specimen in the reference group was incubated from the central axis of the specimen, which is the interface between epoxy and cement mortar. The cracks extended along the interface between epoxy resin and cement until it fractured. The *X*-axis stress cloud map before the crack penetration also showed that the stress sudden change was concentrated on both sides of the interface, indicating that the specimen was destroyed along the interface. As shown in Figure 12b, the specimen with 10% iron tailings filler showed that the fracture crack of the specimen was partially nurtured from the cement mortar and extended diagonally upward along the direction close to the interface fracture. The sudden stress change before penetration was concentrated on the left side of the specimen, that is, the cement mortar part, and the specimen fracture occurred on the side of the cement mortar matrix. In Figure 12c, the fracture crack of the specimen with 20% iron tailings filler was also partially incubated from the cement mortar, and the crack growth showed an irregular stepped shape. The stress mutation before penetration was also unevenly distributed and eventually broke along the cement section. The above phenomenon, combined with the development trend of the bond strength of the specimens, indicated that the iron tailings filler packing inhibited the crack failure of the bond between epoxy mortar and cement mortar, and improved the bond strength of epoxy mortars.

For rapid pavement repair materials, the key performance indicators include high early strength, sufficient compressive and flexural strength, suitable working time, high interfacial bond strength, and low shrinkage. The results obtained in this study show that the epoxy mortar with 20% IT filler meets all the above requirements, indicating its applicability for concrete pavement repair engineering.

The failure modes of flexural bonding specimens, which are categorized into four types (Mode A: failure along the epoxy mortar–cement mortar interface, Mode B: failure on the cement mortar side, Mode C: failure on the epoxy mortar side, and Mode D: mixed failure), are illustrated in Table 6. Failures in the reference group of epoxy resin mortar without filler addition were observed at the interface between the repair mortar and the cement mortar, corresponding to failure Mode D. However, better bonding performance was demonstrated by specimens with iron tailings fillers, with initial fractures occurring on the cement mortar side, indicative of failure Mode B. This finding confirmed that adding iron tailings fillers could enhance the bonding strength of epoxy mortars.

### 3.4. Microstructure and Reinforcement Mechanism of TI Fillers

#### 3.4.1. Morphology by SEM

**Figure 13:**
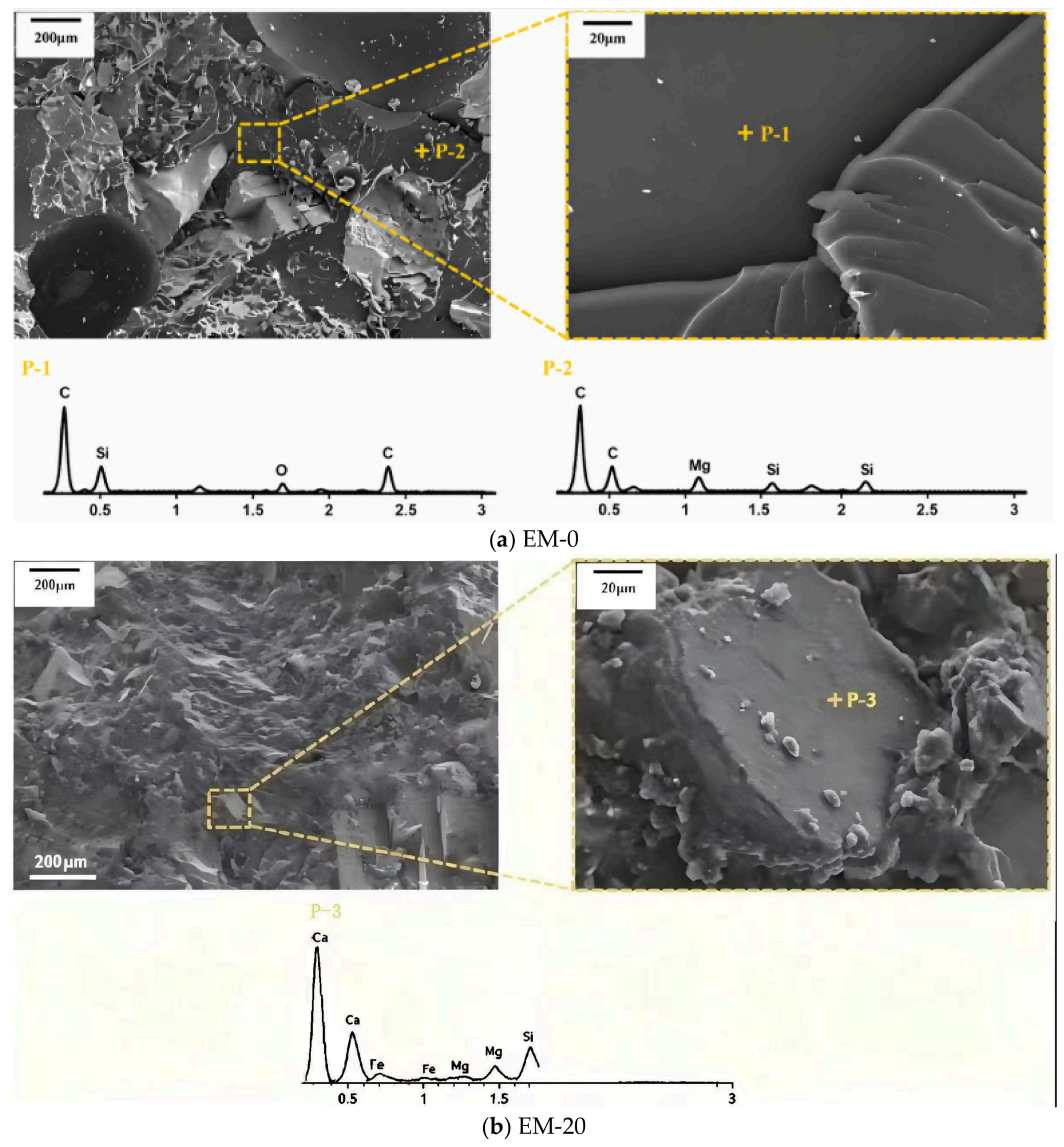

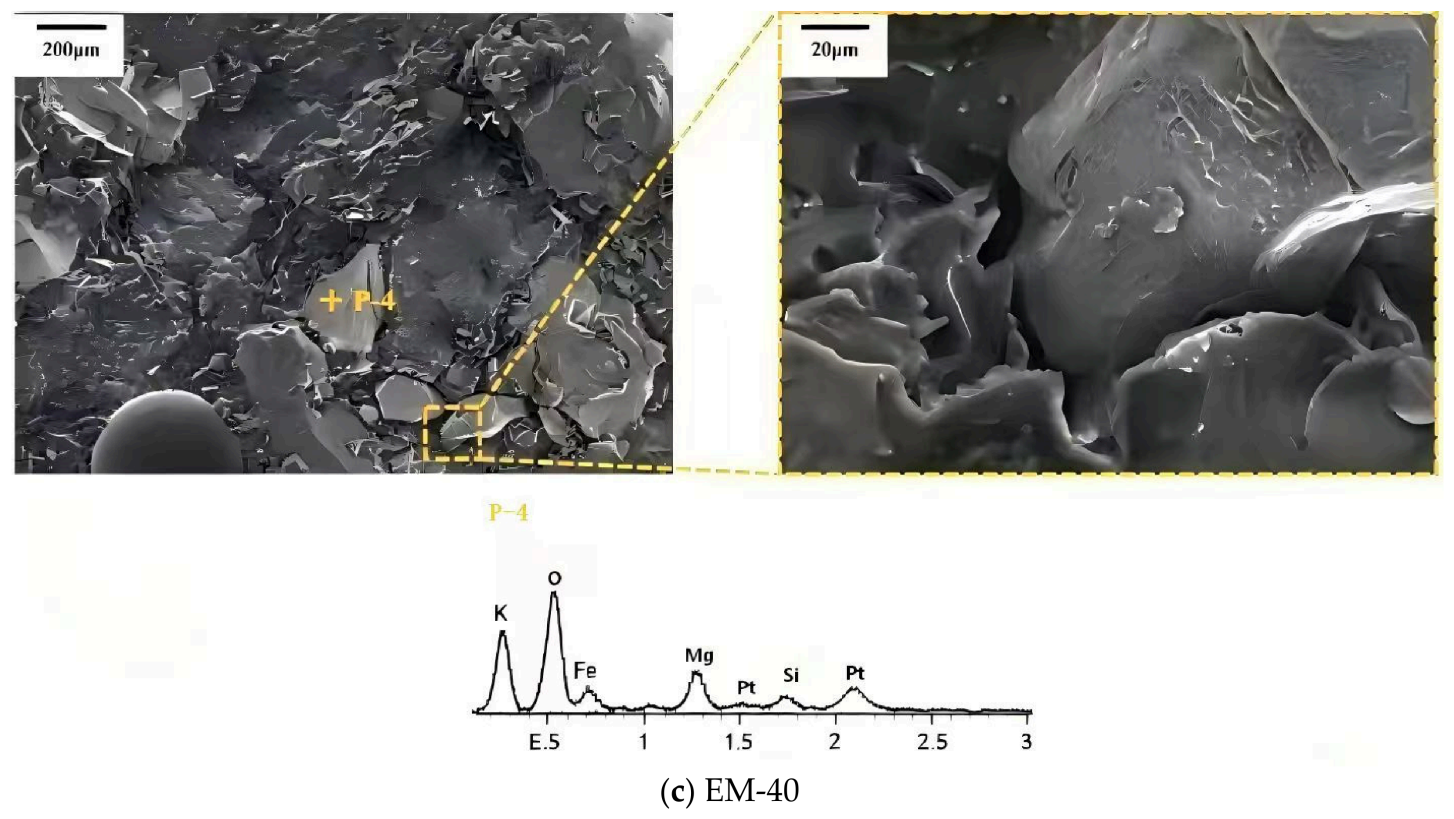
SEM image of fracture surface and EDS analysis of EM with different filler content: (**a**) EM-0, (**b**) EM-20, (**c**) EM-40.

To further investigate the mechanism of how iron tailings fillers and fine aggregates affect the properties of epoxy mortar, scanning electron microscope (SEM) images of the control group EM-0 without iron tailings filler, as well as specimens EM-20 and EM-40 with filler contents of 20% and 40%, respectively, were observed as shown in Figure 13. Obvious large voids and cracks were observed on the fracture surface of the epoxy mortar without added iron tailings filler in Figure 13a. The resin surface was relatively flat, indicating that the resin fracture did not encounter significant hindrance. This further confirmed the reason for the poor mechanical properties of the control group mortar. Figure 13b displays the fracture surface of the specimen with 20% iron tailings filler content, where the epoxy resin fracture surface was dense with fewer defects. It could be clearly observed that the resin tightly encased the iron tailings filler particles, indicating that the addition of filler greatly increased the compactness of the mortar. In addition, the resin surface was rougher and uneven compared to the resin surface of the control group, with a stepped appearance. This fracture characteristic was caused by the hindrance of iron tailings filler particles to the propagation of the fracture cracks. The addition of iron tailings filler changed the mode of crack propagation in the resin matrix from uniform and ordered continuous long strips to irregular stepped ones. Consequently, the mortar transformed from brittle failure to ductile failure, greatly enhancing its toughness and strength [44]. Figure 13c portrays the fracture surface of the specimen with 40% iron tailings filler content. It was evident that the dispersion of iron tailings filler particles was not uniform, and there were a few air voids. This was because a large number of particles dispersed in the matrix were prone to agglomeration and could introduce air, resulting in defects and ultimately leading to a decrease in mechanical properties.

#### 3.4.2. Mercury Intrusion Porosimetry Analysis

**Figure 14:**
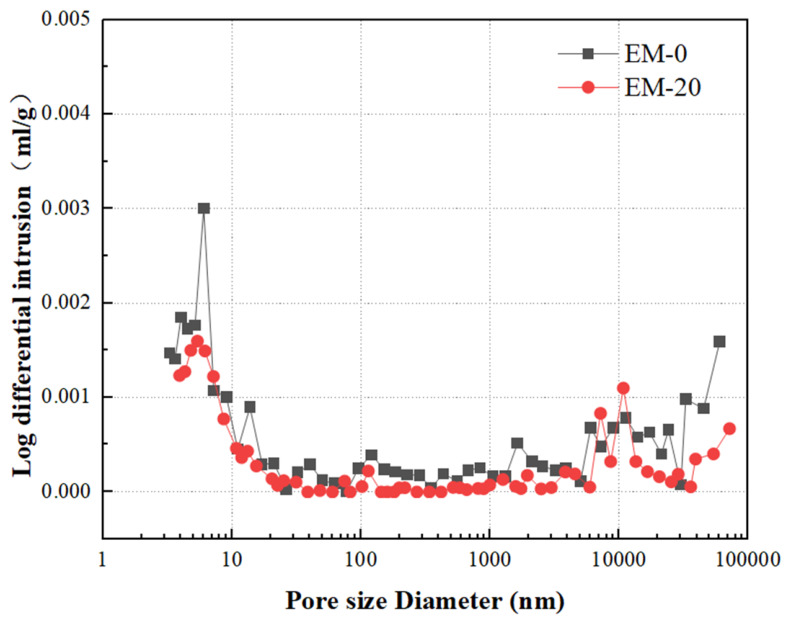
Differential curve of the pore-size distribution of EM under different iron tailings filler content.

The pore structure exerts a decisive influence on the mechanical performance of epoxy repair mortar. Figure 14 presents the differential pore-size distribution curves of specimens with varying iron tailings (IT) filler contents. The blank reference mortar without IT filler exhibited a total porosity of 9.85%, considerably higher than the 4.16% measured for the sample incorporating 20% IT filler. Meanwhile, the reference sample had an average pore diameter of 17.46 nm, larger than the 10.9 nm recorded for the 20% IT-modified epoxy mortar, which demonstrates that the unfilled matrix contains more internal voids.

Pores in all specimens were distributed over two primary ranges: 3–10 nm and 400–100,000 nm. Incorporation of IT filler greatly reduced the volume of macropores ranging from 20,000 nm to 100,000 nm, while moderately increasing pores sized 9000–10,000 nm, which achieves overall pore refinement; nevertheless, the quantity of small pores between 5 nm and 10 nm dropped notably. Such pore evolution stems from the physical filling effect of fine IT particles and improved mortar fluidity, which cuts down trapped air voids inside the matrix and correspondingly enhances mechanical strength. It should be noted that IT filler only refines pores via physical packing and possesses negligible pozzolanic reactivity. In addition, the 20% IT sample (EM-20) delivers far lower porosity than epoxy mortar modified with recycled sand, which confirms the superior grading matching performance of iron tailings when used as fine aggregate and filler.

Mercury intrusion porosimetry (MIP) further reveals that the dominant pore size across all samples lies below 25 nm, falling into the category of harmless gel pores that cannot trigger severe deterioration of mechanical properties. However, as IT dosage continues to rise beyond the optimal 20% proportion, the volume fraction of harmful capillary pores (50–200 nm) rises markedly. Zhao et al. [2] reported the consistent pore evolution trend: higher tailings replacement ratios lead to elevated total porosity and a larger share of medium-to-large pores. This adverse shift at excessive filler dosage can be attributed to two factors: the irregular angular shape of iron tailings weakens inter-particle packing density, and overdosing tailings reduces mixture workability, entraining more air bubbles during mixing. The growing proportion of harmful pores directly explains the decline in compressive strength and interfacial bonding performance of repair mortar, as interconnected capillary pores create localized stress concentration under external load and accelerate microcrack propagation.

#### 3.4.3. The Enhancement Mechanism of IT Fillers on the Epoxy Resin Mortar

**Figure 15:**
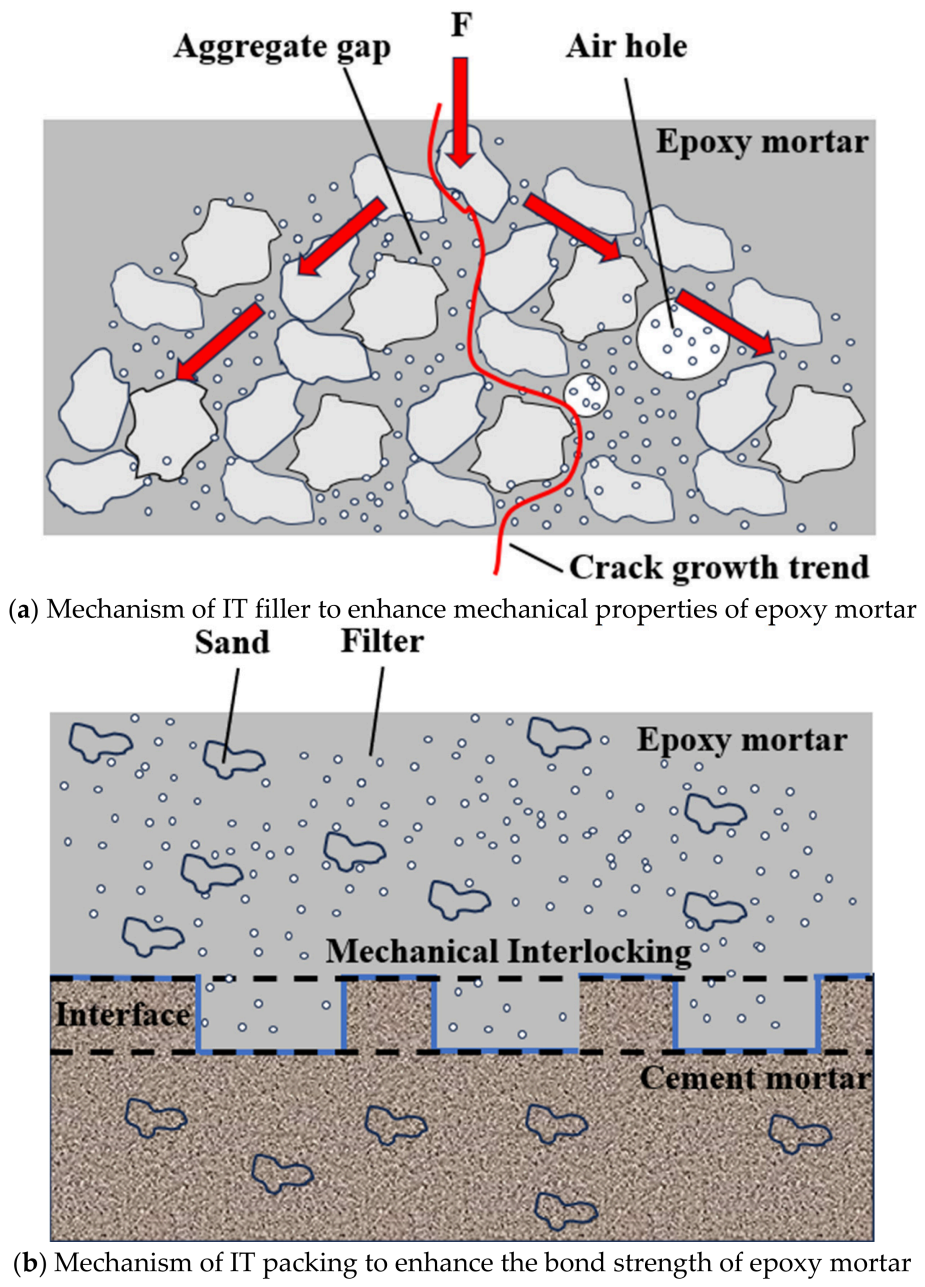
Mechanism of IT filler improving mechanical properties and bonding properties of epoxy mortar.

A mechanistic analysis of how the mechanical properties and bonding performance of epoxy mortar were enhanced by the incorporation of iron tailings filler is presented in Figure 15. As illustrated in Figure 15a, the friction between aggregates was increased by the irregular and angular shape of fine iron tailings aggregates, which was detrimental to mortar flowability. When finer iron tailings fillers were incorporated, the filler particles provided rolling lubrication between aggregates distributed around the aggregates, reducing friction between the aggregates and thereby improving the mortar flowability. Furthermore, the addition of iron tailings fillers filled the pore defects generated during mortar preparation, resulting in a denser mortar, which enhanced its strength and volumetric stability. The fillers allowed the resin and aggregate to form a cohesive entity, which, under external force, enabled load dispersion laterally and hindered crack propagation. Figure 15b elucidates the mechanism by which the bonding strength of epoxy mortar was enhanced by iron tailings fillers. On one hand, the flowability of the epoxy mortar was improved by the incorporation of iron tailings filler, preventing the occurrence of defects at the interface between the epoxy mortar and the substrate, thereby increasing the effective bonding area. On the other hand, the filler addition improved the mortar flowability and wettability, allowing the epoxy resin matrix to penetrate the rough surface and pores of the base mortar, forming a “gripping” mechanical interlock. This increased the bonding area and mechanical interlocking between the epoxy mortar and the old cement mortar, ultimately enhancing the bonding strength of the epoxy mortar.

This phenomenon accords with prior investigations into washed waste functioning as filler [27]. On the one hand, inorganic filler particles fill microvoids and gaps between epoxy matrix and cement hydration products, compacting the microstructure and homogenizing internal stress distribution; on the other hand, rigid fillers pin crack tips during fracture, trigger crack deflection and branching, and extend crack growth paths and consume extra fracture energy, and interfacial debonding and filler pullout further realize energy dissipation and material toughening. The SEM morphologies in Figure 15 intuitively reflect such structural differences, in which unmodified epoxy mortar presents smooth brittle fracture with abundant microvoids, while filler-modified mortar features rugged fracture morphology with fewer pores due to dense packing of fillers.

### 3.5. Engineering Application Prospect

The full-component recycling of iron tailings solid waste has promising application potential. It is expected to cut the consumption of natural quartz sand, lower the raw material cost of repair mortar and ease environmental pollution caused by tailings accumulation. Quantitative verification of cost benefit and environmental benefit needs to be completed through follow-up life-cycle evaluation and cost analysis.

## 4. Conclusions

This study investigated the workability, internal temperature, mechanical properties, and volume stability of epoxy resin mortar prepared with iron tailings as fillers and aggregates. The experimental results yielded the following findings:

(1) Iron tailings are feasible to be fully reused as dual-functional aggregate and filler for pavement repair epoxy mortar. With the filler content increasing from 0% to 40%, the flowability of fresh mortar rises by 34.5 mm with an increment of 24.24%, and the setting process is significantly accelerated.
(2) The mechanical strength and interfacial bonding strength of modified mortar follow a typical trend of first improvement and then deterioration, and the optimal filler dosage is determined as 20%. At this mixing proportion, the 28 d compressive strength reaches 113.2 MPa, the flexural strength reaches 42.5 MPa, and the 7 d interfacial bond strength reaches 9.1 MPa.
(3) Moderate incorporation of IT filler optimizes the pore-size distribution inside the matrix, densifies the internal microstructure and inhibits crack propagation under external loads, which effectively improves the structural stability of hardened epoxy mortar. Excessive IT filler triggers severe particle agglomeration, introduces harmful voids and interrupts the continuous crosslinking network of epoxy resin, thereby weakening the overall performance of composites.
(4) The failure mode of the bonding interface changes from pure interfacial debonding to cohesive failure of the concrete substrate, indicating strong interfacial interaction between the prepared mortar and pavement base.

More comprehensive statistical analysis will be performed in follow-up studies. Relevant durability indicators critical for repair materials, such as Ultraviolet (UV) resistance, freeze–thaw resistance, chemical corrosion resistance, and long-term service performance, will be further investigated in future research.

## Figures and Tables

**Table 1:** Technical specifications of E51 epoxy resin.

Item	Epoxy Equivalent/(g·mol^−1^)	Viscosity/(mPa·s, 25 °C)	Density/(g/cm^3^)	Volatile Content/%	PH
Parameter range	184~195	7000~18,000	1.10~1.20	≤0.5	6~7

**Table 2:** Technical specifications of curing agent 593.

Item	Epoxy Value/(eq/100 g)	Color/APHA	Viscosity/cps	Organic Chlorine/(eq/100 g)	Inorganic Chlorine/(eq/100 g)	Initial Boiling Point/°C
Specification	≥0.43	≤30	100~200	≤0.02	≤0.0005	180

**Table 3:** Technical specifications of diluent 692.

Item	Color (APHA)	Viscosity (25 °C)	Epoxy Value/(eq/100 g)	Saponifiable Chlorine/%	Inorganic Chlorine/(eq/100 g)	Water Content/%
Performance index	≤20	4–10	0.45~0.48	≤0.3	≤20	≤0.1

**Table 4:** Chemical composition of iron tailings (%).

Al_2_O_3_	CaO	K_2_O	MgO	Fe_2_O_3_	SO_3_	SiO_2_
14.48	1.59	2.37	3.32	26.88	3.96	46.30

**Table 5:** Mix design of EM.

Sample	Epoxy Resin E51/g	Curing Agent 593/g	Diluent 692/g	IT Fillers/g	IT Fine Aggregates/g
EM-0	100	30	15	0	500
EM-10	100	30	15	50	450
EM-20	100	30	15	100	400
EM-30	100	30	15	150	350
EM-40	100	30	15	200	300

**Table 6:** Failure modes in flexural bond test.

**Specimen Group**	**Failure Modes in Flexural Bond Test**		
	**1 d**	**7 d**	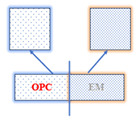 Mode A: Bonding interface failure 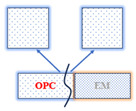 Mode B: Cohesive failure in OPC 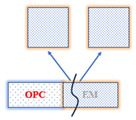 Mode C: Cohesive failure in EM 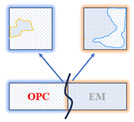 Mode D: Mixed failure
EM-0	D	D	
EM-10	B	B	
EM-20	B	B	
EM-30	B	B	
EM-40	B	B	

## Data Availability

The original contributions presented in this study are included in the article. Further inquiries can be directed to the corresponding authors.
